# Editorial: Hallmark of cancer: avoiding immune suppression

**DOI:** 10.3389/fonc.2023.1211456

**Published:** 2023-06-16

**Authors:** Faris Q. B. Alenzi, Benedetta Apollonio, Liusheng Peng, Elias J. Sayour, Michal Sheffer

**Affiliations:** ^1^ Prince Sattam Bin Abdulaziz University, Al-Kharj, Saudi Arabia; ^2^ S.S.D. Rare Tumors and Melanoma, IRCCS Istituto Tumori “Giovanni Paolo II”, Bari, Italy; ^3^ Department of Microbiology and Biochemical Pharmacy, College of Pharmacy, Third Military Medical University, Chongqing, China; ^4^ Department of Neurosurgery, University of Florida, Gainesville, FL, United States; ^5^ Dana–Farber Cancer Institute, Boston, MA, United States

**Keywords:** immunosuppression, metabolomics, tumor microenvironment, cancer immunotherapy, prediction

Immunosuppression remains a dominating force in the refractory nature of most malignancies. Unlocking immunotherapeutic efficacy requires a more detailed understanding of the cancer ecosystem, including neovascularization, hypoxia patterns, tumor metabolomics, stromal architecture and immunobiology. This understanding is critical to making more informed decisions regarding evolutionary biology, cancer cell senescence, and the tumor immune microenvironment and may be used to predict treatment strategies. In this Research Topic, papers are collated that focus on cancer-mediated immunosuppression as a hallmark. The review by Santiago-Sanchez et al. highlights the myriad of mechanisms and pathways implicated in cancer-mediated immunosuppression. The scope of work in the collection ranges from imaging and radiogenomics in ovarian cancer, to long-noncoding RNA prediction in hepatocellular carcinoma, and glucose metabolism and response to anti-PD-1 therapy in papillary thyroid cancer. Additional articles for this hallmark pertain to hypoxia and machine learning in lung adenocarcinoma, alternative splicing in bladder cancer while others focus on specific molecules of interest including SIGLEC15 in thyroid cancer, TIGIT expression in solid tumors, pyroptosis gene TP63 in osteosarcoma and HSP90 expression in lymphoma to inform immunotherapeutic application. As newer immunotherapy approaches continue to be developed, so must our understanding of the immune landscape across malignancies to inform rationale predictions and treatment modalities. The papers herein reflect a breadth of tools that could be used to make informed decisions in distinct cancer subsets. With the boom of artificial intelligence and public repositories harboring genomic and transcriptome data sets, it is conceivable that more accurate predictions regarding individual cancer biology will be made to inform treatment. The degree, scope and cell types involved in immunosuppression must be better delineated for cancer immunotherapy to meet its promise. The work herein helps add to the growing chorus of tools that will allow for more informed decisions regarding individual cancer immunobiology and provide renewed hope that immunotherapy may be leveraged as an adaptable tool for patients based on their biology for more personalized intervention.

## Author contributions

All authors listed have made a substantial, direct, and intellectual contribution to the work and approved it for publication.

